# Nitrofurantoin—Microbial Degradation and Interactions with Environmental Bacterial Strains

**DOI:** 10.3390/ijerph16091526

**Published:** 2019-04-30

**Authors:** Amanda Pacholak, Wojciech Smułek, Agnieszka Zgoła-Grześkowiak, Ewa Kaczorek

**Affiliations:** 1 Institute of Chemical Technology and Engineering, Poznan University of Technology, Berdychowo 4, 60-695 Poznan, Poland; amanda.d.pacholak@doctorate.put.poznan.pl (A.P.); wojciech.smulek@put.poznan.pl (W.S.); 2 Institute of Chemistry and Technical Electrochemistry, Poznan University of Technology, Berdychowo 4, 60-965 Poznan, Poland; agnieszka.zgola-grzeskowiak@put.poznan.pl

**Keywords:** nitrofurantoin, pharmaceutical, biodegradation, microbial strains isolation, cell membrane permeability, cell metabolic activity

## Abstract

The continuous exposure of living organisms and microorganisms to antibiotics that have increasingly been found in various environmental compartments may be perilous. One group of antibacterial agents that have an environmental impact that has been very scarcely studied is nitrofuran derivatives. Their representative is nitrofurantoin (NFT)—a synthetic, broad-spectrum antibiotic that is often overdosed. The main aims of the study were to: (a) isolate and characterize new microbial strains that are able to grow in the presence of NFT, (b) investigate the ability of isolates to decompose NFT, and (c) study the impact of NFT on microbial cell properties. As a result, five microbial species were isolated. A 24-h contact of bacteria with NFT provoked modifications in microbial cell properties. The greatest differences were observed in *Sphingobacterium thalpophilum* P3d, in which a decrease in both total and inner membrane permeability (from 86.7% to 48.3% and from 0.49 to 0.42 µM min^−1^) as well as an increase in cell surface hydrophobicity (from 28.3% to 39.7%) were observed. Nitrofurantoin removal by selected microbial cultures ranged from 50% to 90% in 28 days, depending on the bacterial strain. Although the isolates were able to decompose the pharmaceutical, its presence significantly affected the bacterial cells. Hence, the environmental impact of NFT should be investigated to a greater extent.

## 1. Introduction

**Figure 1:**
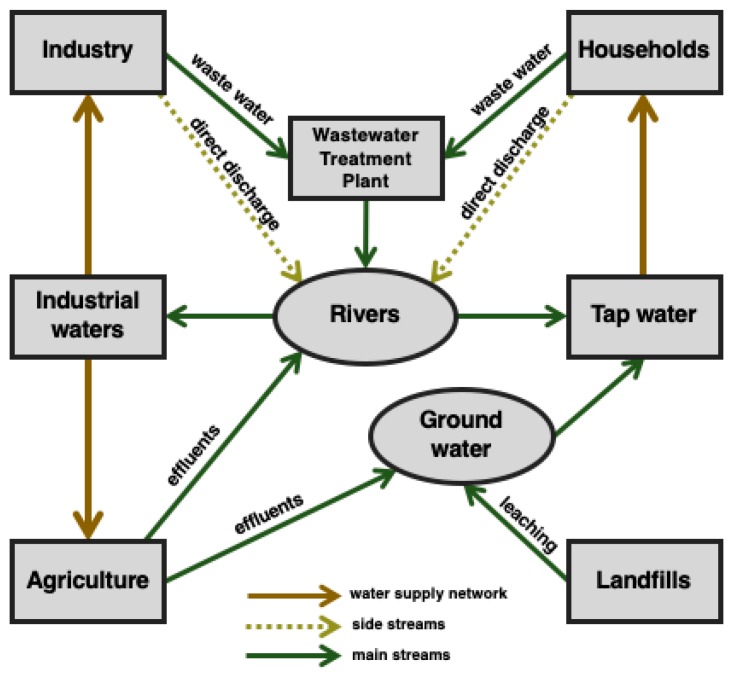
Sources of natural environment contamination with pharmaceuticals. Side and main streams indicate the relative amount of pharmaceutical contaminants introduced to the environment.

Being increasingly used by millions of peoples, pharmaceuticals have become an indispensable element of contemporary societies [1,2,3]. Many of them are purchased and consumed in quantities that exceed the real demand, and recent studies have shown that these substances are entering the natural environment. There are many ways that medicines make their way into the environment (Figure 1). These include improper drugs disposal or the inappropriate management of drug manufacturing facilities. Nevertheless, a great portion of the pharmaceuticals that are present in ecosystems comes from the drugs taken by people or animals that are excreted in an unchanged form in urine or feces [2,4,5]. 

Although the concentrations of pharmaceuticals detected in the environment usually do not exceed safety regulations, their possible adverse impact on fauna and flora cannot be neglected. The particularly dangerous group of compounds found in the environment are antibiotics [6]. The social concerns about them are related not to their concentration in the environmental compartments, but rather to the nature of the molecules and their biological activity [4,6]. The continuous, low-level, unintentional exposure of living organisms and microorganisms to antibiotics contribute to, among other factors, the development of pathogenic bacteria resistance to these compounds [7]. Antibiotics present in the environment also affect natural microbial communities. A number of research studies have demonstrated that the presence of antibacterial pharmaceuticals influences the microbial growth, enzymatic activity, and biomass production, leading to the decrease in microbial diversity [8,9,10,11,12,13,14]. On the other hand, through being involved in the self-purification process of ecosystems and being able to decompose the xenobiotics, natural bacterial communities play a key role in the environmental fate of antibiotics [1,15]. By far, a considerable number of antibiotics has been tested according to their biodegradability. However, one group of antibacterial compounds whose environmental impact has been very scarcely studied is nitrofuran derivatives. They belong to synthetic, broad-spectrum antibiotics that are active against Gram-positive and Gram-negative bacteria. The most often used nitrofuran compound is nitrofurantoin (NFT): an imidazolidinedione derivative containing a distinctive 5-nitrofuran ring [7]. Its bacteriostatic and bactericidal effects are complex and not entirely understood, but might be related to the inhibition of bacterial DNA, RNA, and protein synthesis as well as the formation of reactive oxygen species [16,17,18]. 

Nitrofurans used to be applied in livestock production; however, according to their possible carcinogenic properties, its usage in the fields in question has been prohibited in the European Union since 1995. Nevertheless, they are still easily accessible and used in the treatment of urinary tract infections in humans as well as in veterinary medicine [17,19]. 

Since studies about the environmental effects of nitrofurantoin as well as its biodegradation are limited [20,21], the main aim of this study is to investigate the ability of environmental microbial strains to decompose nitrofurantoin and study the impact of the compound mentioned on microbial cell properties. The analyses performed include the isolation of new bacterial strains from rural and municipal activated sludge as well as their biochemical characterization. Afterwards, the biodegradation of nitrofurantoin by newly isolated microbes was studied with the use of HPLC-MS/MS. Moreover, microbial cell viability and changes in the inner and total membrane permeability as well as cell surface hydrophobicity after contact with nitrofuran-derived drugs were tested. To the best of our knowledge, this is the first comprehensive report on the impact of nitrofurantoin on environmental microbial strains.

## 2. Materials and Methods

### 2.1. Chemicals

For preparing all the media and aqueous solutions, ultra-purified Mili-Q water (Arium^®^ Pro, Sartorius, Kostrzyn Wlkp., Poland) was used. The chemicals applied in the experiments, e.g., nitrofurantoin, 3-(4,5-dimethylthiazol-2-yl)-2,5-diphenyltetrazolium bromide, salts, and medium components, were of the highest purity grade (98% or greater). They were purchased from Sigma-Aldrich (Poznan, Poland). 

### 2.2. Isolation and Identification of Bacterial Strains

**Figure 2:**
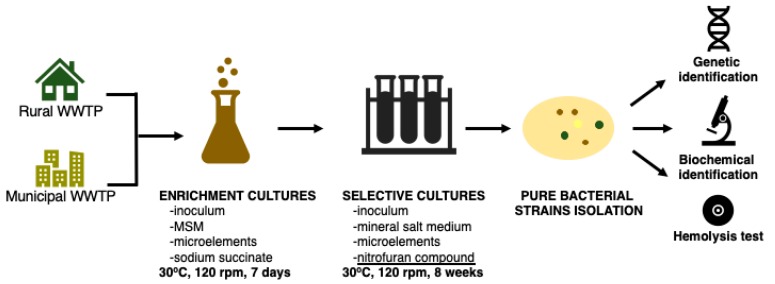
Isolation of microorganisms from the activated sludge—scheme of the procedure.

The bacterial strains that are able to grow in the presence of nitrofurantoin were isolated from activated sludge samples collected aseptically from the rural waste water treatment plant (WWTP) located in Kaźmierz, Poland (52°29′41.6″ N, 16°35′08.8″ E) serving small households, and from the WWTP in Poznań, Poland (52°25′53.1″ N, 16°57′31.8″ E) collecting sewage from the municipal agglomeration. After incubation for 24 h at 30 °C, 10 mL of the activated sludge was transferred to 90 mL of sterile mineral salt medium containing 1 mL of aqueous solution of sodium succinate (20%) and nitrofurantoin (1 mg mL^−1^). Every 7 days, the microorganisms were transferred to a fresh culture medium. The amount of nitrofurantoin was successively increased (by 1 mg L^−1^ every week for five weeks; the last three weeks, it was maintained at 5 mg L^−1^) and sodium succinate content was decreased (by 0.125 mL every week). As a consequence, after 8 weeks, the only carbon source was nitrofurantoin at a concentration of 5 mg L^−1^. In the next step, 0.1 mL of the cultures were seeded on Mueller–Hinton agar medium plates (bioMerieux, Warsaw, Poland), and after 24 h of incubation, a streaking technique was used to isolate the colonies formed by individual bacterial strains. After the isolation, the strains that showed the best growth in the presence of NFT were selected and identified by 16S rRNA gene sequencing in accordance with the method described in our previously published article [22]. Figure 2 reports the main isolation steps. 

### 2.3. Biochemical Characterization of Bacterial Strains

**Figure 3:**
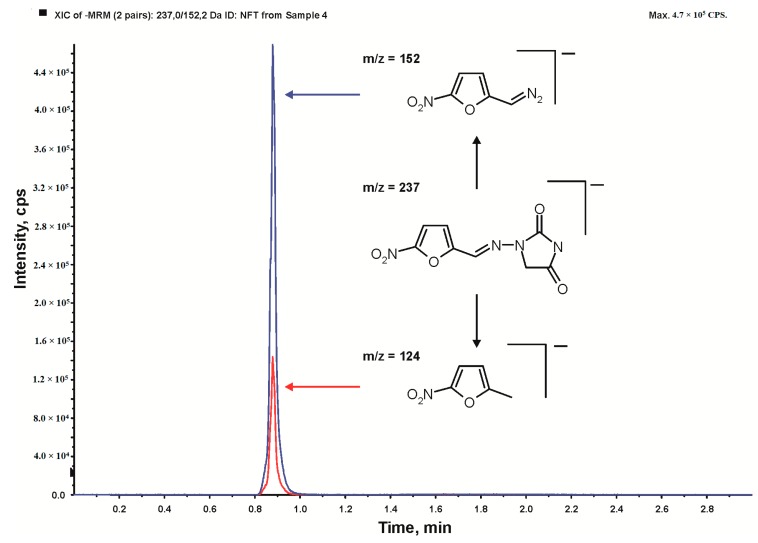
A typical nitrofurantoin chromatogram with two fragmentation transitions used in the multiple reaction monitoring mode.

In order to characterize the pure bacterial strains isolated from municipal and rural WWTPs, their biochemical profiles were evaluated using the Vitek 2 Compact (bioMerieux, Warsaw, Poland) system. Moreover, all the isolated strains were inoculated on plates with Columbia agar containing 5% sheep blood (bioMerieux, Warsaw, Poland). After incubation for 24 h at 30 °C, the color and transparency of the agar medium around the bacterial colonies were observed. These observations can indicate the strains’ ability to cause the lysis of red blood cells, which may be caused by the production of surface-active compounds secreted outside the cell by bacteria [23,24]. The main isolation and characterization steps are depicted in Figure 3. 

### 2.4. Growing Conditions

Isolated and characterized pure bacterial strains were stored on tryptic soy agar plates at 4 °C. They were subcultivated every 21 days. 

The liquid bacterial cultures used in the experiments contained 45 mL of nitrofurantoin solution prepared in mineral salt medium (2.8 mg dissolved in 500 mL of medium under sterile conditions), 5 mL of inoculum, 0.1 mL of sodium succinate (20% aqueous solution), and 0.1 mL of trace elements solution. The composition of the mineral salt medium (MSM) (composition (g L^−1^): Na_2_HPO_4_∙2H_2_O 7.0, KH_2_PO_4_ 2.8, NaCl 0.5, NH_4_Cl 1.0) included the addition of sodium succinate and microelements (MgSO_4_∙7H_2_O 0.35, FeSO_4_∙7H_2_O 0.035, CuSO_4_∙7H_2_O 0.2, MnSO_4_∙5H_2_O 0.2, ZnCl_2_ 0.105, CoSO_4_∙7H_2_O 0.025, H_3_BO_3_ 0.285).

The inocula that were used to prepare the liquid cultures were prepared by adding the loop full of cells taken from an agar plate to sterilized nutrient broth. Such mixtures were incubated at 30 °C with shaking at 120 rpm (KS 4000 ic control, IKA Werke GmbH, Staufen, Germany) for 24 h. Afterwards, the bacteria were centrifuged at 4000× *g* for 10 min (3–18K, Sigma Laborzentrifugen GmbH, Osterode am Harz, Germany) and washed twice with mineral salt medium. Finally, the cell pellet was re-suspended in sterile medium to reach the final bacteria concentration of 1∙10^9^ cfu mL^−1^ (mid log phase; optical density (OD_600_) 1.0 at λ = 600 nm; spectrophotometer Jasco V-650, Tokyo, Japan).

The microbial cultures were incubated at 30 °C with shaking at 120 rpm over a period of time depending on the experiments (biodegradation, 28 days; cell viability and cell surface properties, 24 h). They were conducted in the sterile 250-mL Duran^®^ Schott (Wertheim, Germany) laboratory glass bottles. All the solutions and glassware were sterilized prior to use in the experiments. In order to prevent contamination, the laminar flow cabinet was used during activities associated with biological samples.

### 2.5. Cell Membrane Permeability and Cell Surface Hydrophobicity

In order to evaluate the influence of the presence of NFT (initial concentration 5 mg L^−1^) on microbial strains isolated from rural and municipal activated sludge, analyses of microbial inner membrane permeability, total membrane permeability, and cell surface hydrophobicity were performed. The methods applied includedo-nitrophenyl-β-D-galactoside assay (ONPG), crystal violet assay (CV), and congo red assay (CR). Microbial cultures with and without the addition of NFT were established as described in Section 3.3. Control samples contained mineral salt medium instead of NFT solution. After 24 h, microbial cultures were centrifuged (4000× *g*, 10 min) and washed with mineral salt medium. Afterwards, they were resuspended in MSM to obtain the OD_600_ = 1.0. The total reaction volume was 1 mL. Inner membrane permeability was tested as described previously [25] by measuring the concentration of β-galactosidase released into the solution using ONPG as a substrate. Total membrane permeability was tested according to [26] by colorimetric measurements of the uptake of crystal violet solution by microbial cells (CV assay). Cell surface hydrophobicity was analyzed by measuring the adsorption of congo red dye on the surface of microbial cells (CR assay) [27]. 

### 2.6. Microbes Viability after Contact with NFT

Evaluation of microbial cells’ viability after 24-h contact between microbes and nitrofurantoin as well as control samples (cultivated in the same manner but without the addition of NFT) was performed using 3-(4,5-dimethylthiazol-2-yl)-2,5-diphenyltetrazolium bromide assay (MTT) in accordance with the method described in [28]. After 24 h, microbial cultures were centrifuged (4000× *g*, 10 min) and washed with mineral salt medium. Afterwards, they were resuspended in MSM to obtain OD_600_ values ranging from 0.1 to 0.2. The research samples contained 0.9 mL of microbial suspension and were incubated with 0.1 mL of 5 g L^−1^ MTT solution (the final concentration of MTT in the samples was 0.5 g L^−1^) for 2 h at 30 °C. After incubation, the cultures were centrifuged at 15,000× *g*. The supernatant was discarded, and the pellet (the formazan precipitate formed by viable cells) was dissolved with 1 mL of propan-2-ol. Afterwards, the samples were centrifuged again at 4000× *g*, and the supernatant was analyzed on a UV-Vis spectrophotometer at 560 nm.

### 2.7. Nitrofurantoin Biodegradation with Kinetic Study 

The biodegradation of nitrofurantoin by bacterial strains isolated from rural and municipal sewage was analyzed in the present study. Microbial cultures were prepared as described in Section 2.4. In order to determine the content of residual nitrofurantoin in microbial cultures, the samples were taken in 3 to 4-day intervals. 

The qualitative and quantitative analysis of residual nitrofurantoin was analyzed using HPLC/MS-MS. The chromatographic system UltiMate 3000 RSLC from Dionex (Sunnyvale, CA, USA) was used. Five-µL samples were injected into a Gemini-NX C18 column (100 mm × 2.0 mm i.d.; 3 μm) from Phenomenex (Torrance, CA, USA) maintained at 35 °C. The mobile phase employed in the analysis consisted of ammonium acetate (5 10^−3^ mol L^−1^) in water and methanol at a flow rate of 0.3 mL min^−1^. Gradient elution was performed by linearly increasing the percentage from 75% methanol to 80% in 2 min, and then linearly increasingthe percentage to 100% in 1 min. The LC column effluent was directed to the API 4000 QTRAP triple quadrupole mass spectrometer from AB Sciex (Foster City, CA, USA) through the electrospray ionization source operating in the positive ion mode. The dwell time for each mass transition detected in the MS/MS multiple reaction monitoring mode was set to 200 ms. All the ions were detected using the following settings for the ion source and mass spectrometer: curtain gas, 10 psi; nebulizer gas, 40 psi; auxiliary gas, 40 psi; temperature, 400 °C; and collision gas, medium. The ion spray voltage was −4500 V, and the declustering potential was −60 V. The multiple reaction monitoring transitions parameters were as follows: analytical m/z = 237 → m/z = 152 (collision energy 17 eV, collision cell exit potential 10 V), confirmatory m/z = 237 → m/z = 124 (collision energy 20 eV, collision cell exit potential 10 V); see Figure 3.

Moreover, the nitrofurantoin biodegradation study was extended with kinetic analysis. According to Bekins et al. [29], zero-order calculations, first-order calculations, and the simplified Monod equation were used to calculate the biodegradation kinetics.

### 2.8. Statistical Analysis

All the results are reported as mean values calculated from at least three independent experiments. The statistical significance of differences between the means of research samples and control samples (without the addition of NFT) were determined by one-way analysis of variance (ANOVA) with Tukey’s range test applied as posthoc analysis. Differences with *p* < 0.05 were considered statistically significant. The calculations were performed using Statistica v13 (StatSoft, Cracow, Poland). 

## 3. Results and Discussion

### 3.1. Isolation and Identification of Microbial Strains

The microorganisms used in our research were isolated from samples of activated sludge (AS) taken from municipal and rural waste water treatment plants (WWTPs). In order to isolate pure bacterial strains, selective liquid cultures were established and inoculated with a given sludge sample. After eight weeks of cultivation, 18 bacterial strains were isolated from the rural AS, and 13 were isolated from the municipal one. Thereafter, the strains that showed the best growth in the presence of NFT were selected and identified by 16S rRNA gene sequencing. From the rural WWTP, only two strains were able to use the NFT as the only carbon and energy source, and they were identified as *Sphingomonas paucimobilis* (K3a) and *Ochrobactrum antrophi* (K3b). Both strains are often found in activated sludge [30,31], and are recognized as efficient biodegraders of biocides such as triclocarban (*Sphingomonas* strain described by [32]), triclosan (two *Sphingomonas* strains investigated by [33]), oxytetracycline (*Ochrobactrum* sp. KSS10 studied by [34]), sulfamethoxazole (*Ochrobactrum* sp. SMX-PM1-SA1 isolated by [35]), or erythromycin (*Ochrobactrum* sp. described by [36]). In municipal WWTP activated sludge, three strains showing the capability of degrading nitrofurantoin were found: *Rhizobium radiobacter* (P4c), *Pseudomonas aeruginosa* (P4a), and *Sphingobacterium thalpophilum* (P3d). *R. radiobacter* has been previously recognized as degrading several dyes [37,38]; the second strain is an ubiquitous environmental bacteria displaying great biodegradation potential [39,40]. However, the strain from the *Sphingobacterium thalpophilum* genus was found in activated sludge fed with pharmaceutical waste water [41].

Furthermore, for all the isolated strains, the biochemical profile was evaluated using a Vitek2^®^ system with a GN Colorimetric Identification Card. The results of differentiating reactions are presented in Table 1. The Vitek2^®^ system (bioMerieux, Warsaw, Poland) provides information about 48 characteristic biochemical reactions. Among them, 23 were common for all the investigated strains. The strains belong to different genera, and the variety of biochemical profiles is comprehensible. However, L-proline arylamidase and tyrosine arylamidase assimilation were observedin all the strains. 

The next step of the microbial strains’ characterization was the analysis of their ability to produce hemolysins. For that puropse, microbial strains were grown on Columbia agar containing 5% sheep blood (bioMerieux, Warsaw, Poland) using the streaking technique. Among the tested strains, only *P. aeruginosa* (P4a) caused the complete lysis of the blood cells (β-hemolysis). However, the lysis could be observed in the place of the massive growth of bacteria only. Presumably, the amount of hemolysins produced by the strain was too small to cause the rupture of red blood cells where the single colonies were formed. Moreover, *O. antrophi* (K3b) displayed α-hemolysis because the agar under the colony was dark and greenish. The agar under the colonies of *S. paucimobilis* (K3a), *R. radiobacter* (P4c), and *S. thalpophilum* (P3d) was unchanged. It means that those strains did not induce hemolysis (i.e., were non-hemolytic).

### 3.2. Inner and Total Membrane Permeability

**Figure 4:**
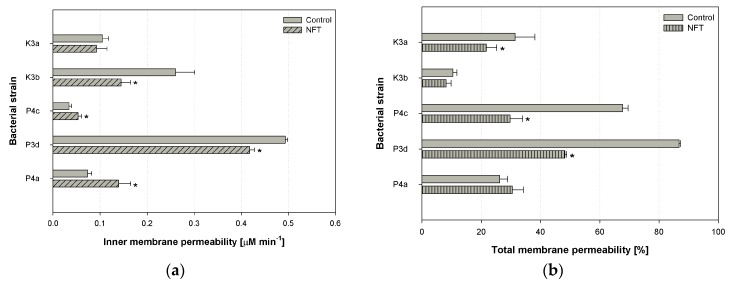
Changes in inner membrane permeability (**a**) and total membrane permeability (**b**) in five microbial strains subjected and not subjected to contact with nitrofurantoin; *Sphingomonas paucimobilis* (K3a), *Ochrobactrum antrophi* (K3b), *Rhizobium radiobacter* (P4c), *Pseudomonas aeruginosa* (P4a), and *Sphingobacterium thalpophilum* (P3d); stars (*) above the columns indicate statistical differences among groups (samples treated with nitrofurantoin (NFT) vs. untreated controls, *p* < 0.05, ANOVA followed by Tukey’s range test); results are reported as mean values calculated from three independent experiments.

The experiments performed within the study included the analysis of inner and total membrane permeability. The parameters were tested in cells subjected to 24-h contact with nitrofurantoin (initial concentration 5 mg L^−1^), and those that were cultivated under the same conditions but without the addition of NFT. All the experiments were performed in independent quadruplicates. Afterwards, the statistical analysis was performed, and statistically significant differences were indicated. The results of experiments are depicted in Figure 4. 

One-way analysis of variance (ANOVA) and further posthoc tests showed that nitrofurantoin did not cause any modifications in the inner membrane permeability of the K3a strain (*p* = 0.992054) (Figure 4a). The average value of the parameter tested was 0.10 μM min^−1^. However, statistically significant differences in inner membrane permeability were observed in microbial cells of strains K3b, P4c, P3d, and P4a between control samples and samples with NFT. The strongest difference was observed in K3b and P3d strains (both *p*-values < 0.001). In both strains, the permeability of the inner membrane was lower after contact with NFT. A different situation was observed in the cells of the P4c and P4a strains: permeability was significantly greater in the microbial cultures with nitrofurantoin compared to the culture that contained mineral salt medium only. Increases from 0.03 to 0.05 μM min^−1^ and from 0.07 to 0.14 μM min^−1^ were observed. What is more, within the five microbial strains tested, P4c was characterized by the smallest inner membrane permeability, and P3d had the greatest.

The results of the inner membrane permeability measurements obtained within the present study are similar to those of Guven et al. (2005), who investigated the effect of various antibiotics and pesticides on the inner membrane permeability of *E. coli* ML 35 using the same method as in the present article. Their experiments revealed that neither gramicidin D nor ampicillin modify the activity of the enzyme β-galactosidase in the ML 35 strain [42]. This means that the presence of those antibiotics did not influence the permeability of the ML 35 membrane, which is similar to how nitrofurantoin did not modify the permeability of the K3a and P4c strains. On the other hand, Rajasekaran et al. (2019) have performed the experiments on antimicrobial peptides and their analogs. They checked, among other factors, the β-galactosidase activity in the *E. coli* strain after contact with the peptides. They observed an increase or no change in the membrane permeability of the inner membrane [43]. In our studies, nitrofurantoin induced a statistically significant increase in the permeability of the inner membrane in only one (P4a) of the five strains.

Figure 4b depicts the results of modifications of the total membrane permeability after the addition of nitrofurantoin (these groups are described as ‘NFT’). The greatest differences between the control samples and samples containing nitrofurantoin were observed in the P4c and P3d strains (*p* < 0.001 for both strains). The presence of NFT provoked a reduction in total membrane permeability by 40% of the microbial cells mentioned. A statistically significant modification in total membrane permeability was noticed also for the K3a strain (a decrease from 31.4% to 21.7%, *p* = 0.038384). However, no changes were observed in the case of K3b (*p* = 0.996302) and P4a (*p* = 0.838065). Interestingly, the same direction of changes in both inner and total membrane permeability was observed for P3d (a statistically significant decrease in the presence of NFT) and P4a (a statistically significant increase in the presence of NFT), as well as K3a and K3b (a decrease in the presence of NFT); however, for the last two strains, the modifications were not significant for at least one parameter. Moreover, it should be highlighted that among all the strains tested, *S. thalpophilum* (P3d) was characterized by the greatest permeability change for both the bacterial total and inner membrane (in both the ‘Control’ and ‘NFT’ samples). 

In general, the contact of bacteria cells with antimicrobial agents often causes an increase in the total membrane permeability. For example, ciprofloxacin and rhamnolipids provoked an increase of crystal violet uptake by *S. aureus* and *E. coli* cells [44]. Similarly, Bharali et al. (2013) observed rhamnolipids enhancing the membrane permeability of *K. pneumonie* cells [45]. However, ranbezolid, the pharmaceutical containing a nitrofuran ring in its molecule, did not affect *S. aureus* membrane integrity, but rather strongly damaged the membrane of *S. epidermidis* [46].

Most often, the increase of cellular membrane permeability is related to toxic effect of the xenobiotics on the cell and leads to its rupture [47]. On the other hand, it can facilitate the uptake of these compounds, and can be useful in biodegradation processes, as observed in the research of Qiu et al. [48]. Moreover, it should be mentioned that the permeability of the bacterial inner membranes decreased after contact with NFT in the K3b and P3d strains, and the permeability of the total membrane was statistically reduced in the K3a, P4c, and P3d strains. These results can suggest that the bacteria initiated a cellular defense mechanism against the exogenous substance and tried to prevent the xenobiotics molecules from entering into the cell [49].

### 3.3. Cell Surface Hydrophobicity

**Figure 5:**
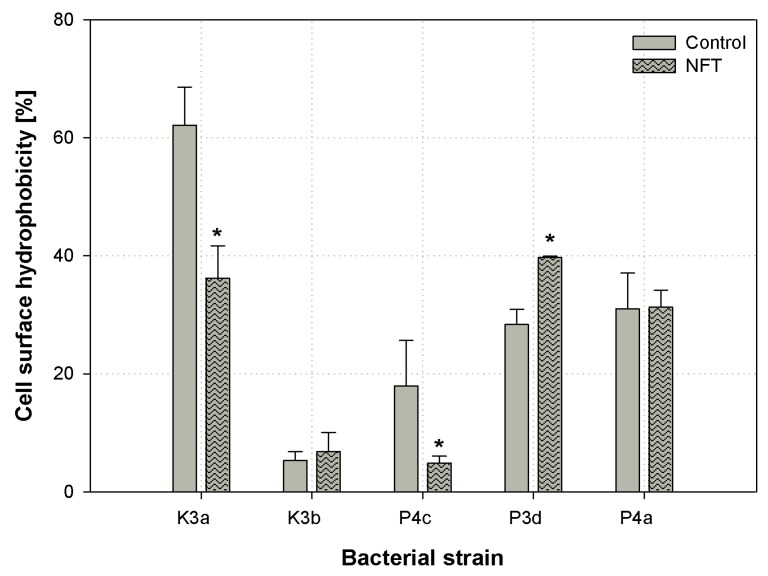
Changes in cell surface hydrophobicity in five microbial strains subjected and not subjected to contact with nitrofurantoin: *Sphingomonas paucimobilis* (K3a), *Ochrobactrum antrophi* (K3b), *Rhizobium radiobacter* (P4c), *Pseudomonas aeruginosa* (P4a), and *Sphingobacterium thalpophilum* (P3d); stars (*) above the columns indicate statistical differences among groups (samples treated with NFT vs. untreated controls, *p* < 0.05, ANOVA followed by Tukey’s range test); the results are reported as mean values calculated from three independent experiments.

An important parameter of the bacterial cell surface that may indicate the chemical compound bioavailability to microbial cells is the cell surface hydrophobicity (CSH). In our research, the CSH was tested using colorimetric congo red assay [27]. The results are presented in Figure 5. Among all the strains tested, *S. paucimobilis* (K3a) was characterized by the highest CSH (62.1%), as measured in the control samples. The presence of NFT provoked a decrease in the hydrophobicity of K3a to 36.2% (*p* < 0.001). A statistically significant reduction in the parameter in question was also noticed in P4c; however, an increase in CSH was observed in P3d. Considering K3b and P4a, no significant changes in the cell surface hydrophobicity were investigated. However, it should be noted that the cells of K3b were characterized by strongly hydrophilic properties (CSH between 5.3–6.8%), but the P4a cells were slightly hydrophobic (CSH around 31.3%).

The carbon source may strongly affect the cell surface hydrophobicity. In turn, the bioavailability of the compound to be degraded may be increased [50]. However, the number of factors influencing the cell surface hydrophobicity impedes the easy interpretation of the mechanism of modification of bacteria cells’ surface properties [51]. The alteration in cell surface hydrophobicity is clearly noticeable in the presence of hydrophobic pollutants such as diesel oil [52]. What is important, the pharmaceuticals, such as ibuprofen, also can modify the hydrophobicity of the bacterial cell surface [53]. Similarly, the presence of nitrofurantoin provoked changes in the CSH of the bacteria tested. It may indicate remodeling the outer layers of bacterial cells and changes in substrate bioavailability. However, phenomena occurring during the assimilation of a degraded compound are complex, and modifications of CSH do not have to directly affect changes in biodegradability.

### 3.4. Cytotoxity Analysis of NFT

**Figure 6:**
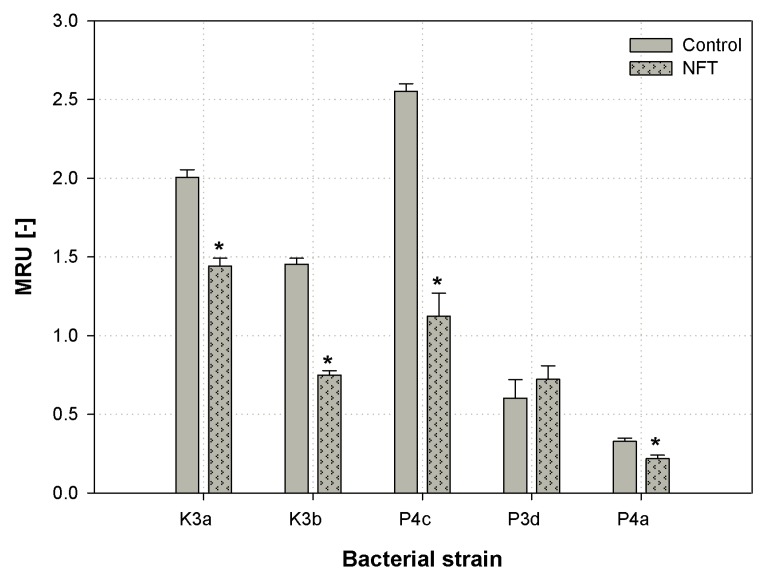
Modifications of microbial cells’ viability in microbial cultures subjected and not subjected to contact with nitrofurantoin after 24 h of cultivating: *Sphingomonas paucimobilis* (K3a), *Ochrobactrum antrophi* (K3b), *Rhizobium radiobacter* (P4c), *Pseudomonas aeruginosa* (P4a), and *Sphingobacterium thalpophilum* (P3d); stars (*) above the columns indicate statistical differences among groups (samples treated with NFT vs. untreated controls, *p* < 0.05, ANOVA followed by Tukey’s range test); results are reported as mean values calculated from three independent experiments.

Microbial cells viability is an important parameter during the evaluation of the xenobiotic’s toxicity. In the present paper, the MTT (3-(4,5-dimethylthiazol-2-yl)-2,5-diphenyltetrazolium bromide) method was used in order to observe the effect of nitrofurantoin presence on the metabolic activity of newly isolated microbial strains [28]. The results are presented as MTT reducing units (MRU) in Figure 6.

The one-way ANOVA test and Tukey’s range test indicated that nitrofurantoin provoked a significant decrease in the cell metabolic activity of strains K3a, K3b, P4c, and P4a (*p*-value < 0.001, all four strains). The strongest difference was observed for P4c, where the relative number of metabolically active cells decreased from 2.55 to 1.12 MRU [-]. However, the slightest decrease that was still significant was noticed for the P4a strain. Considering the microbial strain isolated from the municipal WWTP, *S. thalpophilum* P3d, no notable changes in cells metabolic activity were noticed between the sample with MSM and the one with NFT. It should be noticed that a slight increase in metabolic activity was noticed in the P3d strain after contact with NFT; however, the difference was not significant (*p* = 0.614344).

Zhang et al. (2013) investigated the effect of another nitrofuran derivative—furazolidone—on microbial metabolic activity. The results showed that the relative cytotoxicity of the compound in question ranged from 6% to 36%, regarding the bacterial strain tested [54]. Another study published by Bergheim et al. (2015) showed that antibiotics are highly toxic to the *Pseudomonas putida* strain. High toxicity was associated with the low degradability of those compounds [3]. Such correlation did not have a place in our research. Although the presence of antimicrobial exogenous compounds decreased cells’ viability, its biodegradation by the strains tested was still efficient (see Section 3.5). Itis interesting that the highest decrease in cells’ metabolic activity (P4c) was not observed in the strain that had the smallest degradation rate (P4a). 

### 3.5. Nitrofurantoin Removal

**Figure 7:**
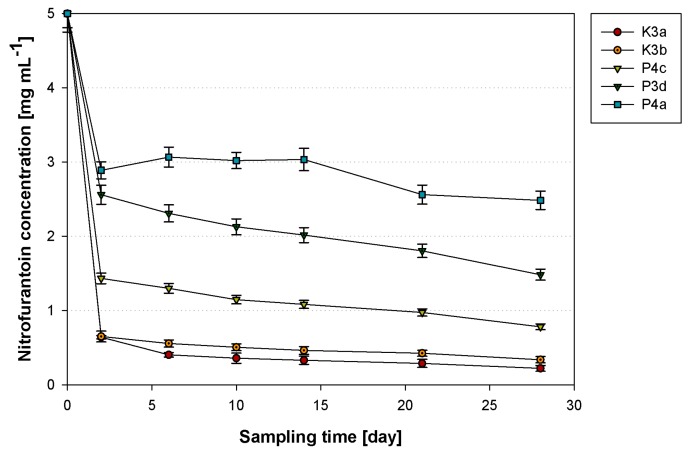
The nitrofurantoin removal in cultures with strains *Sphingomonas paucimobilis* (K3a), *Ochrobactrum antrophi* (K3b), *Rhizobium radiobacter* (P4c), *Pseudomonas aeruginosa* (P4a), and *Sphingobacterium thalpophilum* (P3d); results are reported as the mean values calculated from three independent experiments.

The next stage of the research was devoted to the analysis of the degradation potential of nitrofurantoin by the isolated bacterial strains. The highest removal rate was observed for all the strains within the first two days of cultivation (Figure 7). However, microorganisms from the rural WWTP, *S. paucimobilis* K3a and *O. antrophi* K3b, displayed the highest nitrofurantoin removal efficiency. Both strains utilized nearly 90% of the initial amount of the pharmaceutical in two days. At the end of the experiments, on the 28th day, the concentration of the degraded compound was reduced to 0.22 mg L^−1^ and 0.34 mg L^−1^ in cultures with K3a and K3b, respectively. Analyzing the results obtained for the strains of municipal WWTP origin, the most effective degradation was conducted by the P4c strain. The concentration of nitrofurantoin was reduced to 0.78 mg L^−1^ in four weeks (84% of the initial NFT concentration was utilized). At the same time, the concentration of NFT measured in cultures containing P3d and P4a on the 28th day was 1.48 and 2.49 mg L^−1^, which corresponded to 70% and 50% removal efficiency, respectively.

An important aspect of nitrofurantoin degradation is its relation to microbial cell properties. Reorganizations of cellular structures are expected to depend on bacterial species. As described above, microbial strains identified as *S. paucimobilis* K3a and *O. antrophi* K3b exhibited the greatest degradation ability. However, the response of these bacterial cells to the presence of nitrofurantoin was equivocal. We observed that in K3a, a high degradation was accompanied by the modification of the cells surface toward hydrophilic properties, and total membrane permeability toward significantly lower values. Nevertheless, the permeability of the inner membrane has not been changed. A different situation was noticed in the case of K3b, where a high degradation rate was accompanied by a significant decrease in inner membrane permeability, and the other parameters (total membrane permeability and cell surface hydrophobicity) have not been changed. On the other hand, the lowest nitrofurantoin removal rate was observed for *P. aeruginosa* P4a. In this case, the contact of bacterial cells with nitrofurantoin induced a statistically significant increase in both the inner and total membrane permeability. It is interesting that among all the strains tested, P4a was the strain that was characterized by the lowest metabolic activity, which was additionally reduced after cells’ exposure to nitrofurantoin. The strain in question was also the only one that exhibited β-hemolysis. The results obtained indicate a variety of changes in the properties of the tested strains. Different observations can be explained by the different mechanisms of uptake of the substrate.

The information about the biodegradation of nitrofurantoin and other nitrofuran-derived compounds is scarce. Among the articles published in the last decade, only [54] studied furazolidone biodegradation by bacterial strains *Acinetobacter calcoaceticus* T32, *Pseudomonas putida* SP1, and *Proteus mirabilis* V7. On the second day of the experiment, the concentration of the compound in the cultures decreased significantly, and was lower than 30% of the initial concentration. Moreover, it is worth mentioning that Samuelsen et al. (1991) have analyzed the impact of furazolidone on microorganisms from aquaculture sediments, and noted that the half-life of furazolidone (at initial concentration of 400 μg mL^−1^) did not exceed 18 h. However, an addition of the compound tested strongly reduced the number of autochthonic bacteria in the sediment [55]. 

Table 2 presents the results of an NFT degradation kinetic study calculated for individual microorganisms. The calculations results accurately match the Monod model, which allowed determining the main constants in an equation describing the above-mentioned kinetic model. A half-saturation constant (*K_s_*) representing the calculated substrate concentration at which its biotransformation is half the maximum value had the highest value for the P4c, P3d, and P4d strains (2.87, 3.68, and 4.09 mg L^−1^, respectively). In contrast, the lowest values did not exceed 2.63 mg L^−1^, and were found for two strains of rural WWTP origin. The distribution of substrate utilization rate (*v_m_*) values among the tested bacteria was relatively similar to the *K_s_* distribution. Microorganisms from the municipal activated sludge exhibited relatively low utilization rates (0.01 mg L^−1^ day^−1^). The strains from the rural activated sludge had higher values of *v_m_*: 0.02 and 0.06 mg L^−1^ day^−1^ for K3b and K3a, accordingly. 

The kinetics of antimicrobial agents’ biodegradation have been analyzed by several researchers; however, this has never been studied in relation to nitrofurantoin. What is more, the results of antibiotics biodegradation have not always fitted the Monod model. For example, the sulfadiazine biodegradation by *Arthrobacter* spp. followed first-order decay kinetics [56]. In contrast, Cheyns et al. [57] after an analysis of atrazine biodegradation suggested that the Monod equation fits better. Hence, the obtained results are important, because the proper kinetics model can help predict the biodegradation capacity of microorganisms [57,58].

## 4. Conclusions

Both microbial consortia used (municipal and rural WWTPs’ activated sludge) contained microbial strains that were capable of using NFT as a source of carbon and energy. Among the microbes isolated, the best nitrofurantoin removal efficiency was displayed by *S. paucimobilis* K3a and *O. antrophi* K3b (on average, 90% of the initial concentration of NFT was reduced in 28 days). Nitrofurantoin induced a decrease in both theTMP and CSH of the K3a strain. In general, the microorganisms that had their permeability lowered in the presence of NFT were characterized by lower cell metabolic activity. 

## Figures and Tables

**Table 1:** The differentiating reactions from Vitek2^®^ GN Colorimetric Identification Card (bioMerieux, Warsaw, Poland) for strains coming from the rural WWTP: *Sphingomonas paucimobilis* (K3a), *Ochrobactrum antrophi* (K3b) and the municipal wastewater treatment plant (WWTP): *Rhizobium radiobacter* (P4c), *Pseudomonas aeruginosa* (P4a), and *Sphingobacterium thalpophilum* (P3d).

Strain	APPA	ILATk	GlyA	O129R	ADO	dMAL	dTAG	AGLU	PyrA	AGLTp	dTRE	SUCT
K3a	+	+	−	−	−	+	−	+	+	−	−	−
K3b	−	+	+	−	+	−	+	−	+	−	−	+
P4c	−	−	+	−	−	−	−	−	+	−	−	−
P4a	−	+	−	+	−	−	−	−	−	+	(+)	−
P3d	−	−	−	−	−	−	−	+	−	−	−	−
	IMLTa	IARL	dGLU	dMNE	CIT	ELLM	GGT	URE	MNT	CMT	BAlap	BGUR
K3a	−	−	+	−	+	−	−	+	−	−	−	−
K3b	−	−	−	−	−	+	−	+	−	−	+	−
P4c	−	+	−	−	−	+	−	+	−	−	−	−
P4a	+	−	+	+	+	−	+	−	+	+	+	−
P3d	−	−	−	−	−	−	−	+	−	−	−	+

APPA—Ala-Phe-Pro-arylamidase; ILATk—L-lactatealkalinisation; GlyA—glycinearylamidase; O129R—O/129 resisitance (comp. vibrio); ADO—adonitol; dMAL—D-maltose; dTAG—D-tagatose; AGLU—alpha-glucosidase; PyrA—L-pyrrolydonyl-arilamidase; AGLTp—glutamylarylamidasepNA; dTRE—D-trehalose; SUCT—succinatealkalinisation; IMLTa—L-malateassimilation; dMNE—D-mannitol; CIT—citrate (sodium); ELLM—Ellman; GGT—gamma-glutamyl-transferase; URE—urease; MNT—malonate; CMT—coumarate; BAIap—β-alaninearylamidasepNA; BGUR—β-glucoronidase.

**Table 2:** The kinetics parameters according to the Monod equation describing nitrofurantoin biodegradation by strains *Sphingomonas paucimobilis* (K3a), *Ochrobactrum antrophi* (K3b), *Rhizobium radiobacter* (P4c), *Pseudomonas aeruginosa* (P4a), and *Sphingobacterium thalpophilum* (P3d).

Bacterial Strain	Half-Saturation Constant K_s_ [mg L^−1^]	Substrate Utilization Rate v_m_ [mg L^−1^ day^−1^]	R^2^
K3a	2.13	0.02	0.99
K3b	2.63	0.06	0.99
P4c	2.87	0.01	0.99
P3d	3.68	0.01	0.99
P4a	4.09	0.01	0.99
